# Regulatory complexity revealed by integrated cytological and RNA-seq analyses of meiotic substages in mouse spermatocytes

**DOI:** 10.1186/s12864-016-2865-1

**Published:** 2016-08-12

**Authors:** Robyn L. Ball, Yasuhiro Fujiwara, Fengyun Sun, Jianjun Hu, Matthew A. Hibbs, Mary Ann Handel, Gregory W. Carter

**Affiliations:** 1 The Jackson Laboratory, Bar Harbor, ME USA; 2 Department of Computer Science, Trinity University, San Antonio, TX USA

**Keywords:** Meiosis, Spermatogenesis, Maximum covariance analysis, Mouse, Transcriptome, RNA-seq

## Abstract

**Background:**

The continuous and non-synchronous nature of postnatal male germ-cell development has impeded stage-specific resolution of molecular events of mammalian meiotic prophase in the testis. Here the juvenile onset of spermatogenesis in mice is analyzed by combining cytological and transcriptomic data in a novel computational analysis that allows decomposition of the transcriptional programs of spermatogonia and meiotic prophase substages.

**Results:**

Germ cells from testes of individual mice were obtained at two-day intervals from 8 to 18 days post-partum (dpp), prepared as surface-spread chromatin and immunolabeled for meiotic stage-specific protein markers (STRA8, SYCP3, phosphorylated H2AFX, and HISTH1T). Eight stages were discriminated cytologically by combinatorial antibody labeling, and RNA-seq was performed on the same samples. Independent principal component analyses of cytological and transcriptomic data yielded similar patterns for both data types, providing strong evidence for substage-specific gene expression signatures. A novel permutation-based maximum covariance analysis (PMCA) was developed to map co-expressed transcripts to one or more of the eight meiotic prophase substages, thereby linking distinct molecular programs to cytologically defined cell states. Expression of meiosis-specific genes is not substage-limited, suggesting regulation of substage transitions at other levels.

**Conclusions:**

This integrated analysis provides a general method for resolving complex cell populations. Here it revealed not only features of meiotic substage-specific gene expression, but also a network of substage-specific transcription factors and relationships to potential target genes.

**Electronic supplementary material:**

The online version of this article (doi:10.1186/s12864-016-2865-1) contains supplementary material, which is available to authorized users.

## Background

Spermatogenesis is a complex developmental process with a unique cell division, meiosis, as a major defining event. The entire process includes maintenance of a small population of spermatogonial stem-cells, mitotic divisions of differentiating spermatogonia, meiotic prophase and ensuing divisions of spermatocytes, and post-meiotic differentiation of spermatids, by a process known as spermiogenesis. In mammalian testes, spermatogenesis occurs within seminiferous tubules, where all germ cells associate with one kind of somatic cell, the Sertoli cell, which provides the appropriate niche and microenvironment for the spermatogenic process. The adult testis is characterized by presence of all of the cells types in the spermatogenic lineage, with waves of differentiation throughout the testis propelled by retinoic acid signaling [1]. In mice, the first wave of spermatogenesis is initiated by spermatogonia shortly after birth, producing a sequential and orderly appearance of each of the more differentiated stages at regular intervals though the first four weeks of life. Although semi-synchronous with respect to the advancing wave of the most differentiated cells, the juvenile onset of spermatogenesis also includes regular and asynchronous initiation of subsequent waves of spermatogenic differentiation. This asynchronous and continuous process of spermatogenesis has made it difficult to achieve molecular characterization of specific cell types in the lineage. This has been particularly the case with respect to analysis of the defining process of gametogenesis, meiosis, which occurs in spermatocytes. The complex events of meiosis I prophase include recombination, homologous chromosome pairing, and synapsis, taking place as spermatocytes progress through the leptotene, zygotene, pachytene and diplotene substages. These events culminate in the first meiotic division, a reductive division in which homologous chromosomes are separated, producing secondary spermatocytes that rapidly undergo the second, equational, meiotic division to produce haploid round spermatids. Because of the genetic importance of meiotic recombination for the production of chromosomally normal gametes and offspring, there has been great interest in elucidating the molecular hallmarks and their underlying transcriptional signatures that define the meiotic spermatocyte substages.

Toward the goal of achieving a molecular understanding of spermatogenesis, considerable effort has been devoted to separation of specific germ-cell differentiation substages from the histologically complex seminiferous epithelium. A widely used approach, commonly known as the “STA-PUT” sedimentation process [2–4], involves enzymatic dissociation of germ and somatic cells and enrichment for specific stages by cell-size-based sedimentation at unit gravity on a bovine albumin gradient. Reasonably good enrichment of the most uniquely sized cells (large pachytene spermatocytes and small round spermatids) can be obtained from testes of adult mice. However, many cells of interest (spermatogonia, early meiotic prophase spermatocytes) are not retrieved from adults because of their anatomical position bounded by Sertoli cell tight junctions. While these early cell types can be retrieved from juvenile testes, the amount of biological material required is daunting and enrichment is not robust. Isolation of specific cell types by fluorescence-activated cell sorting, FACS [5–8] is promising and becoming more widely applied; however sample sizes are small. Finally, rather than cell separation, total testis germ-cell populations can be collected in a developmental continuum during the first two to three weeks of juvenile development of mouse testes, to take advantage of the first wave of spermatogenesis. In this way, molecular features have been defined with respect to the sequential appearance of more advanced cell stages of spermatogenesis. This approach is useful primarily for correlating the appearance of a molecular entity to the developmental appearance of a specific cell type. However, the degree of resolution has been suboptimal, because gene or protein expression has not been related to absolute frequencies of cell stages, a challenge we tackle in this study.

Together, these methods for enriching or inferring spermatogenic (and meiotic) substages have contributed to studies over the past decade on the developmental transcriptome of mammalian spermatogenesis, as recently reviewed [9]. Most such studies have taken advantage of microarrays querying known coding sequences [6, 10–12], and thus sequence-biased, but also provided interesting views of alternative splicing and other features of the spermatogenic transcriptome [13], and identified previously unknown potential targets for contraception and fertility [14]. More recently, methods for unbiased high-throughput deep sequencing of the transcriptome by RNA-seq have been employed [5, 15–19]. These studies have revealed unappreciated regulation of piRNAs [17], and global whole-genome views of the male germ-cell transcriptome [5, 18, 19]. However, the challenge for all transcriptomic analyses, particularly RNA-seq approaches, has been to computationally deconstruct the entire testis or germ-cell transcriptome into substage-specific transcriptomes. This is an important goal, given both the abundance and complexity of RNA species in the testis, particularly with respect to the imputed contribution of both coding and non-coding RNA from spermatocytes and spermatids [19]. In one example of such a computational approach, frequencies of specific germ-cell stages throughout the first wave of spermatogenesis previously published in another analysis [4] were used to estimate cell type-specific expression patterns in a separate data set [18]. While coming closer to the goal of substage-specific transcriptomes, this study relied on a low sample size and on integrating non-contemporaneous data. Other approaches [5, 6], employed cell sorting by FACS and subsequent validation of purity by meiotic markers. While these studies have yielded important insights into global gene expression switches, they rely on FACS, not always available or practical for small samples, such as from infertile mutant models.

Here we have tackled the problem of deconvolving transcriptomes of complex germ-cell populations into stage-specific transcriptomes by computationally integrating highly detailed and combinatorial cytological staging of the same cell samples as subjected to RNA-seq analysis. Deep and accurate cell stage phenotyping was conducted using antibodies to STRA8, SYCP3, phosphorylated H2AFX, and HISTH1T, all exhibiting meiotic substage specificity of expression and/or localization. By collecting highly enriched germ-cell populations from testes at two-day intervals through the first wave of spermatogenesis, and by multiple sampling of individual mice (30 total) for both cytological composition and RNA composition by high-throughput sequencing, we developed an unusually deep data set that portrays variation in cell substage composition and transcript abundance. To decompose these data into substage-specific transcriptome patterns in an unbiased way, we chose a nonparametric solution with minimal assumptions about data structure, thus developing a novel permutation-based maximum covariance analysis. This method enabled us to assign co-expressed transcripts to one or more meiotic substages, thereby linking distinct molecular programs to cytologically defined cell states. Moreover, to better understand the regulation of each germ-cell substage transcriptome, we integrated transcription factor information to identify some of the key molecular regulators driving these cell stages. This approach provides a model for deconvolving transcriptomes of complex cell populations with well-defined cytological attributes. Together, these data provide an unprecedented view of the complexity of meiotic transcription programs and their coordinate regulation.

## Results

### Experimental design

**Fig. 1:**
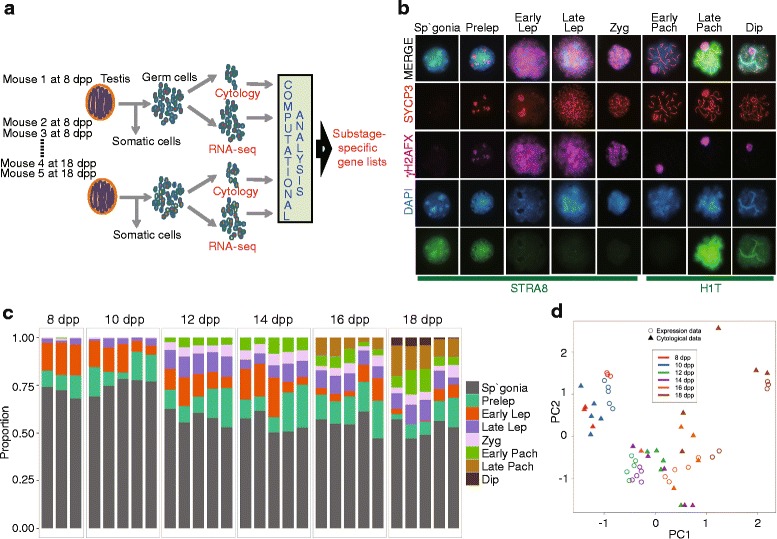
Decomposition of meiotic cells and gene expression. **a** Experimental design. Germ cells were isolated from whole testes from each of five juvenile male mice at 8, 10, 12, 14, 16 and 18 dpp. Each sample was analyzed for both cytology and gene expression by RNA-seq. PMCA was developed to identify the meiotic substage-specific transcriptomes. **b** Cytological classification and cellular populations. Isolated germ cells were immunolabeled for stage-specific maker proteins: STRA8, expressed in differentiating spermatogonia; SYCP3, a component of synaptonemal complex shows meiotic substage-specific labeling morphology; phosphorylated histone H2AFX (γH2AFX), marking DSBs throughout early prophase nuclei and restricted to the XY chromosomes during pachynema and diplonema; and histone HISTH1T, expressed in post-mid-pachytene spermatocytes. Nuclei were counter-stained with DAPI. **c** Meiotic cell composition during the first wave of spermatogenesis. The contribution by specific germ-cell stages for each developmental time point and mouse is shown, with colors representing specific meiotic substages. Abbreviations: Sp’gonia = spermatogonia, Prelep = pre-leptonema, Early Lep = early leptonema, Lep = leptonema, Late Lep = late leptonema, Zyg = zygonema, Early Pach = early pachynema, Late Pach = late pachynema, Dip = diplonema. **d** Concordance of expression and cytological data. Principal component 1 (PC1) versus principal component 2 (PC2) from independent PCA of cytological and RNA-seq data. Colors correspond to time points and icon shape corresponds to data type as indicated

Germ cells were obtained from individual mice at two day intervals from 8 dpp to 18 dpp (*N* = 5 biological replicates at each age; *N* = 30 samples total) and divided into two aliquots, one for cytology and one for RNA-seq (Fig. 1a). For the C57BL/6J (B6) strain used in this study, this developmental window captures the initial differentiation of spermatogonia into early leptonema, through the stages of pachynema and diplonema. For each sample, we determined the relative numbers of cells of each substage by cytological criteria (*N* = about 400 germ cells per mouse, for a total of 11,990 germ cells scored cytologically by criteria described below); by scoring germ cells from each individual mouse, we captured variability between mice. The purity of germ cells in each sample, computed as germ-cell count divided by total DAPI-stained cell count, was greater than 90 % for all samples. Upon inspection of both cytological and RNA-seq data, two germ-cell samples collected at 8 dpp revealed poor concordance with age-matched samples, likely due to insufficient cell numbers for successful RNA-seq library preparation (Additional file 1: Table S1); thus these samples were omitted from all subsequent analyses (final *N* = 28).

### Cytological deconvolution of the meiotic substage composition of germ cells during the first wave of spermatogenesis

Germ-cell substage frequencies in each cytological preparation were determined by combinatorial immunolabeling with antibodies to meiotic substage marker proteins, scoring 400 germ cells per mouse. Marker proteins assessed were STRA8, a meiosis-initiating factor [20] present in differentiated spermatogonia and some leptotene spermatocytes; SYCP3, a synaptonemal complex (SC) protein present in the chromosomal axes of leptotene and zygotene spermatocytes, in the lateral elements of the mature SC in pachytene spermatocytes, and disassembling in diplotene spermatocytes [21, 22]; the phosphorylated form of histone H2AFX (PH2AFX, also known as γH2AX), which localizes to chromatin surrounding DNA double-strand breaks (DSBs) in characteristic patterns that discriminate early meiotic prophase from the pachytene and diplotene stages [22, 23]; and the testis germ cell-specific histone H1 variant HIST1H1T (herein referred to by its common designation of H1T), which is a marker of the mid-to-late pachytene stage [22]. The combinatorial labeling patterns for each marker protein allowed categorization of germ cells of each sample into eight substages, achieving a higher degree of meiotic substage discrimination than previous transcriptome analyses (Table 1 and Fig. 1b).

**Table 1:** Immunolabeling criteria for cell types and meiotic substages

Substages	SYCP3	γH2AX	STRA8	H1T
Spermatogonia^a^	None	Negative	Positive	Negative
Preleptonema	Patches	Negative	Positive	Negative
Early Leptonema	Patches	Positive	Positive	Negative
Late Leptonema	Fine foci	Positive	Weakly positive	Negative
Zygonema	Partially paired	Patches	Negative	Negative
Early Pachynema	Paired	Restricted to XY body	Negative	Negative
Late Pachynema	Paired	Restricted to XY body	Negative	Positive
Diplonema	Partially dissociated	Restricted to XY body	Negative	Positive

^a^Spermatogonia are a separate mitotic stage, not a meiotic substage

Together, these data provide a comprehensive picture of postnatal spermatogenic progress through meiotic prophase of the first wave of differentiating cells for comparison to data previously obtained by histological analyses [4]. At every time point in the current data, greater than 50 % of the retrieved germ cells were spermatogonia (Fig. 1c), reflecting continually initiating separate waves of spermatogenesis. Representation of these cells decreased over time (Fig. 1c), likely due to initiation of additional waves of meiotic cells, increasing numbers of post-spermatogonial spermatocytes, and establishment of cell junctions that impede cell retrieval. Throughout this period, due in part to initiation of subsequent waves of spermatogenesis, the average contribution to the total germ-cell population by preleptotene and leptotene spermatocytes remained relatively constant (Fig. 1c), thus reducing the power to identify transcripts specific to just these substages. The first appreciable numbers of H1T-negative early pachytene spermatocytes were observed at 12 dpp, and H1T-positive late-pachytene cells were abundant by 16-18 dpp, consistent with previously published data [24]. By 18 dpp, less than 10 % of the cells had progressed to the diplotene stage, and only about 0.1 % to metaphase. Overall substage frequencies were similar at 8 and 10 dpp, and also at 12 and 14 dpp (Fig. 1c). For computational analyses (below), we combined substages that exhibited similar frequency patterns across the developmental time span, specifically late leptotene and zygotene substages, and late pachytene and diplotene substages.

### High concordance of gene expression and cytological data

To assess the feasibility of associating gene expression with cytologically-defined substages, we performed independent principal component analyses (PCA) on each data set. The high concordance between the cytological frequency of cell substages and RNA expression data (Fig. 1d) suggested that changes in gene expression in pooled germ cells can be explained by variation in cytological proportions of the differentiation states during spermatogenesis. Although cells in different substages may contribute different amounts of RNA to each sample, this analysis demonstrates an overall quantitative agreement between the two data types. This supported the validity of combining these two datasets to identify meiotic substage-specific transcriptional programs.

### Meiotic substage-specific gene expression derived by covariance analysis

**Fig. 2:**
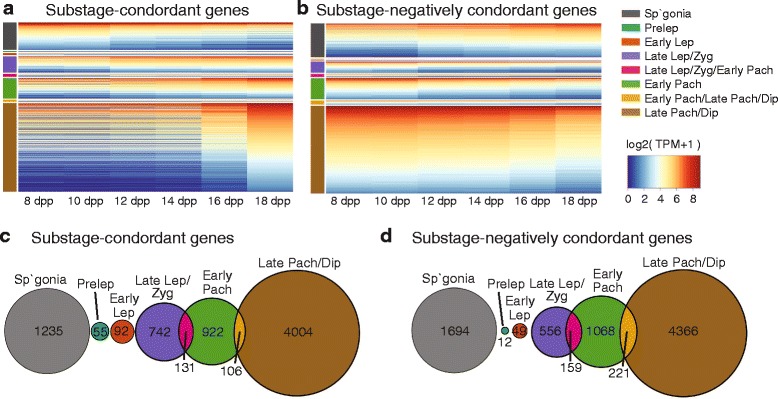
Expression of substage- concordant and negatively concordant genes. **a** Heat maps show gene expression of substage-concordant genes at each time point. **b** Heat maps show gene expression of substage-negatively concordant genes at each time point. Color bars on the left side of the heat maps represent meiotic substages (Abbreviations: Sp’gonia = spermatogonia, Pre Lep = pre-leptonema, Early Lep = early leptonema, Lep = leptonema, Late Lep = late leptonema, Zyg = zygonema, Early Pach = early pachynema, Late Pach = late pachynema, Dip = diplonema). Gene expression is shown as average log2(TPM + 1) over time point replicates. **c** Overlap among substage-concordant gene lists, illustrating the number of transcripts in common between each pair of lists. **d** Overlap among substage-negatively concordant gene lists, illustrating the number of transcripts in common between each pair of lists

To identify signatures for meiotic substage-specific gene expression, we developed a novel permutation-based maximum covariance analysis (PMCA), which maps groups of co-expressed genes to combinatorial cytological marker-based staging based antibodies to STRA8, SYCP3, phosphorylated H2AFX, and HISTH1T. This statistical approach demonstrates *concordance* of distinct cellular programs to each meiotic substage based on the similarity of “cyto-pattern” and “gene-expression pattern” across samples. In brief, the concordant patterns are derived from the most preeminent features of covariance between cytological and transcriptome data across all samples (Methods). In this RNA-seq dataset, a total of 15,025 Ensembl genes (http://www.ensembl.org) and 5267 NONCODE lncRNA genes [25] were detected (at a Transcripts per Million (TPM) ≥ 3) in at least one sample of the isolated germ cells. We also detected piRNA precursors transcripts that had previously been identified [17] with appropriate substages, although they are not the focus in the current study. Through PMCA, we identified 1235 spermatogonia genes and 6052 meiosis substage-specific genes. Expression of many transcripts could not be assigned to distinct substages and instead, were shared across several consecutive substages; 131 genes were shared among late leptotene, zygotene, and early pachytene stages while 106 genes were shared among early pachytene, late pachytene, and diplotene stages (Fig. 2; Additional file 2: Figure S1; Table 2; Additional file 1: Table S2). Notably, increased numbers of expressed genes were observed at 16 dpp when cells first reach the late pachytene stage (Table 2; Additional file 1: Table S2).

**Table 2:** Substage-concordant and negatively concordant genes

Substage	Number of genes	Number of genes
	concordant	negatively concordant
Spermatogonia^a^	1235	1694
Preleptonema	55	12
Early Leptonema	92	49
Late Leptonema/Zygonema	742	556
Late Leptonema/Zygonema/Early Pachynema	131	159
Early Pachynema	922	1068
Early Pachynema/Late Pachynema/Diplonema	106	221
Late Pachynema/Diplonema	4004	4366

^a^Spermatogonia are a separate mitotic stage, not a meiotic substage

In addition to identifying genes concordant with specific substages, PMCA also identified genes that were *negatively concordant* with specific substages. The negatively concordant genes have patterns of expression that are opposite to the cytological patterns; thus negatively concordant defines gene expression that is lower when the cytological proportion of a specific substage is higher and vice versa (Fig. 2; Additional file 2: Figure S2; Table 2; Additional file 1: Table S2). Genes detected as negatively concordant with one stage are often detected as concordant with another substage. For example, genes negatively concordant with spermatogonia are genes expressed across the meiotic cell substages, but not enriched in spermatogonia. Not surprisingly, the 1694 genes negatively concordant with spermatogonia were enriched for known meiotic genes. Indeed, among all genes expressed, many of those with known meiotic function and/or Gene Ontology annotation for meiotic function were not concordant with any single meiotic substage, but were expressed across meiotic substages.

### Validation of substage-specific gene expression patterns

We bolstered and validated these computationally derived results in three distinctly different ways. First, gene expression analyses by qRT-PCR supported the RNA-seq expression data. Second, the validity of the PMCA procedure was queried by analysis of genes expressed in a highly enriched cell population of mid- to late-pachytene spermatocytes retrieved from adult testes by unit gravity sedimentation. Finally, we used our data to confirm a known pattern of male germ-cell specific gene expression, X-chromosome silencing during meiotic prophase.

We compared transcript expression patterns detected by RNA-seq to independent qRT-PCR assays. We tested representative genes among those increasing, decreasing, or showing little change in expression across the sequential time points, as well as genes known to be highly expressed in pachynema. Germ cells of 10 and 16 dpp males were subjected to qRT-PCR to determine expression of each tested gene relative to a reference housekeeping gene (*Actb*); data are shown in Additional file 2: Figure S3. The qRT-PCR assays reflected RNA-seq findings. For example, in 16 dpp samples we found elevated expression of pachytene-enriched genes and of genes shown by RNA-seq to increase in expression throughout the juvenile period tested (Additional file 2: Figure S3). Overall, patterns of gene expression are mostly concordant between the two different quantification methods and between sample sets, although, as expected, RNA-seq provides finer resolution and higher information content.

We further validated the PMCA-derived meiotic substage transcriptomes by comparison to highly enriched adult pachytene spermatocytes obtained by sedimentation at unit gravity [2–4, 26], which sorts cells based on size, not cytology or DNA content (*N* = 4 samples, each from germ cells pooled from testes of 6 mice at 9 weeks of age). The purity of late pachytene/diplotene spermatocytes in this preparation was 90 % based on cytological immunostaining with antibodies to stage-specific proteins phosphorylated H2AFX, SYCP3, and HIST1H1T. RNA isolated from the size-enriched pachytene spermatocytes was subjected to RNA-seq, allowing us to compare genes expressed in the pachytene spermatocytes to the meiotic substage-specific gene lists derived by PMCA. Cross-referencing from this dataset, there was a highly significant enrichment of pachynema-expressed genes among the gene lists from later meiotic substages (hypergeometric tests, all *p* < 2.86 x 10^-18^), but there was no enrichment for pachytene genes in the gene lists for spermatogonia or the early substages of meiosis (Table 3). In fact, 99 % of the genes in the late pachytene/diplotene list were also found in the enriched adult pachytene spermatocyte samples.

**Table 3:** Representation of enriched pachytene spermatocyte genes in meiotic substages

Substage	Number represented/total concordant	*p*-value
Spermatogonia^a^	614/1235	1
Preleptonema	17/55	1
Early Leptonema	28/92	1
Late Leptonema/Zygonema	550/742	1.2 x 10^-09^
Late Leptonema/Zygonema/Early Pachynema	97/131	9.0 x 10^-03^
Early Pachynema	809/922	2.0 x 10^-62^
Early Pachynema/Late Pachynema/Diplonema	102/106	1.3 x 10^-15^
Late Pachynema/Diplonema	3955/4004	<1.0 x 10^-62^

^a^Spermatogonia are a separate mitotic stage, not a meiotic substage

**Fig. 3:**
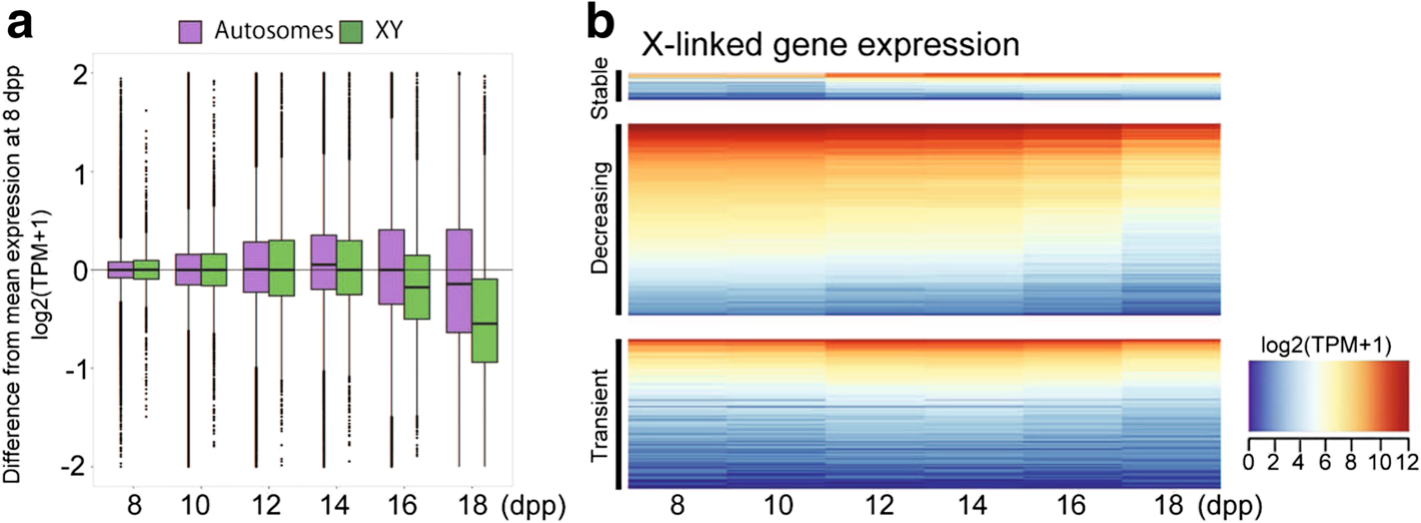
X-linked gene expression during the first wave of spermatogenesis. **a** Boxplots of the difference from mean gene expression at 8 dpp are shown for each time point, with genes on the autosomes in purple and those on the XY chromosomes in green. Gene expression is log2(TPM + 1). **b** Heatmap of X-linked gene expression at each time point. Genes are clustered based on the pattern of expression: an increase of expression at 12 dpp and then stable throughout (Stable), steadily decreasing over time (Decreasing), or low expression from 8-10 dpp and high expression from 12-14 dpp followed by low expression at 16-18 dpp (Transient). Gene expression is shown as average log2(TPM + 1) over time-point replicates

Finally, we queried the meiotic substage-specific patterns of gene expression determined by PMCA by confirming meiotic sex-chromosome inactivation (MSCI), a well-known feature of meiotic gene expression. In spermatocytes, most of the axial length of X and Y chromosomes, which are non-homologous, is not paired or synapsed. By the onset of the pachytene stage of meiotic prophase, the unpaired regions of the sex chromosomes become transcriptionally inactivated [11, 27, 28]. We assessed expression of X-linked genes from our RNA-seq data; overall, 788 X-linked genes (and 24 Y-linked genes) were detected in the substage-associated gene lists. Of these, only 190 genes were detected in the highly enriched adult pachytene spermatocytes, providing evidence of robust MSCI. These 190 transcripts, which do not show any specific regional localization on the X chromosome, might reflect ongoing transcription and escape from MSCI, but, perhaps more likely, may represent stable transcripts persisting after inactivation. In the developmental transcriptome, the strongest X-linked gene signals are those represented in the negatively concordant gene lists (Table 4), that is, genes with expression pattern opposite to the cytologically determined frequencies of each substage. This strong signal is particularly apparent for genes in the late-pachytene negatively concordant list, which exhibit relatively constant expression from 8 to 14 dpp but then sharply decrease in expression at 16 and 18 dpp; this is evidence for pachytene substage MSCI. In addition to MSCI being revealed by the negatively concordant gene lists, there is also diminished representation of X-linked genes in gene lists concordant with later stages of meiotic prophase (Table 4). Overall, 730 X-linked genes appear to be down-regulated by pachynema to late pachynema, or even earlier (Fig. 3a) and only 58 X-linked genes with early expression are detected at later stages (Fig. 3b).

**Table 4:** Substage-specific X-linked gene analysis

Substage	X-linked Concordant	Y-linked Concordant	X-linked Negatively Concordant	Y-linked Negatively Concordant
Spermatogonia^a^	28	0	55	2
Preleptonema	2	0	0	0
Early Leptonema	2	0	1	0
Late Leptonema/Zygonema	34	2	6	0
Late Leptonema/Zygonema/Early Pachynema	3	0	1	0
Early Pachynema	21	1	41	0
Early Pachynema/Late Pachynema/Diplonema	0	0	13	0
Late Pachynema/Diplonema	14	3	329	9

^a^Spermatogonia are a separate mitotic stage, not a meiotic substage

In conclusion, evidence from both MSCI and the transcriptome of highly enriched adult pachytene spermatocytes validates the PMCA-derived substage- transcriptome signatures derived by PMCA. Moreover, as discussed below, we find good concordance among our stage-specific gene lists and functions with those derived by other studies.

### Enrichment analysis of meiotic substage gene expression

The PMCA-derived meiosis substage-specific gene lists, coupled with Gene Ontology (GO) analysis [29], can provide insight into meiotic and spermatogenic function. Genes assigned to each substage were ranked by their similarity score, a measure of how closely the gene’s expression pattern follows the substage’s cytological pattern. Using ranked lists, we performed a ranked GO analysis in GOrilla [30]; these ranked lists provide approximations of meiotic substage gene ontology. Significantly, many meiosis-specific genes are not prominently represented among the meiosis substage-specific gene lists derived by PMCA. However, GO terms for genes negatively concordant with spermatogonia were predominantly meiotic terms (16 out of 31 GO terms for process, all *p* < 9.97 x 10^-4^), confirming that meiotic genes tend to be expressed across multiple meiotic substages and not unique to a particular meiotic substage (Additional file 1: Table S3). Thus meiotic substage transitions are probably not acutely regulated at the level of transcription of many of the known meiotic genes, although among late leptonema/zygonema-associated GO terms, 8 out of 10 GO “biological process” terms were associated with meiosis regulation (all *p* < 8.6 x 10^-4^), and similar GO terms were also identified in the early pachynema gene list (13 out of 33 GO terms for process, all *p* < 5.56 x 10^-4^) (Additional file 1: Table S4). We extracted 447 genes for *M. musculus* that are associated with any GO terms containing “meiosis” or “meiotic” (www.mousemine.org), and of these genes we considered the 404 genes which are expressed in our developmental time series (those genes not expressed in our time series might include female meiosis-related genes). We found that 229 (57 %) genes were not concordant with any specific substage, while 26 (6 %) were concordant with late leptonema and zygonema, 43 (11 %) were concordant with early pachynema, and 92 (23 %) were concordant with late pachynema and diplonema. Of meiotic terms that were negatively concordant with substages, 67 (17 %) were negatively concordant with spermatogonia and 97 (24 %) were negatively concordant with late pachynema and diplonema (Additional file 1: Table S5). GO terms for genes expressed in late meiotic prophase were significantly enriched for spermatogenesis, spermiogenesis and fertilization, reflecting transcription of mRNAs to be stored for later translation during the haploid phase of spermatogenesis [31]. For example, early pachynema gene lists are enriched for GO terms associated with spermiogenesis (5 out of 33 GO terms for process, all *p* < 7.39 x 10^-4^), and the majority of late meiotic prophase- or late-pachynema/diplonema-associated genes were associated with spermiogenesis or fertilization-related GO terms (11 out of 16 GO terms for process, all *p* < 7.11 x 10^-4^) (Additional file 1: Table S4). Although genes concordant with early leptonema had transcription-related GO terms in 11 out of 32 GO terms for process (all *p* < 1.00 x 10^-10^), we found that many genes negatively concordant with leptotene through zygotene substages have GO terms for transcription and related processes (Additional file 1: Table S3), possibly reflecting the cytologically diminished incorporation of RNA precursors during the earliest meiotic prophase substages [32]. Also among the genes lists for these early stages we found extracellular membrane- or molecular transport-related GO terms prominently represented (Additional file 1: Table S4), which may related to the fact that these cells transit through the Sertoli cell junctions that create the blood-testis barrier [33].

Additionally we performed feature enrichment analysis with the hypergeometric test on the substage-specific gene lists. Interestingly, protein-coding genes are enriched in gene lists concordant with the late-leptotene/zygotene stages (*p* = 4.52 x 10^-18^) and the early pachytene stage (*p* = 1.19 x 10^-8^). Moreover, they are enriched in the set of genes that is negatively-concordant with late-pachytene and diplotene substages (*p* = 5.94 x 10^-167^). However, the genes concordant with late-pachytene and diplotene substages are not depleted in protein-coding gene, which comprise the expected majority of this gene set. This suggests that while certain protein-coding genes are abundant in the Late Pach/Dip substage, large numbers of other protein-coding genes are downregulated in this stage, perhaps reflecting cessation of mRNA transcription in preparation for the meiotic division stage, or, alternatively, post-transcriptional regulation by PIWI-interacting RNAs (piRNAs). Recent studies show that piRNAs play important roles in genome stability by suppressing harmful transposons as well as by regulating mRNAs [34], and future analyses could integrate piRNA expression with these data.

### Transcription factor analysis

Because our results point to a large and diverse meiotic germ-cell transcriptome, also noted by others [19], we inferred the underlying regulatory networks accounting for these patterns, using the iRegulon bioinformatic approach to identify transcription factors (TFs) potentially regulating substage-specific genes (Methods). TFs were identified for each substage-specific gene list with high normalized enrichment scores (NES ≥ 4), corresponding to an estimated false discovery rate of less than 0.01 [35]. We then determined which TF genes were unique to, or shared among, the substages. As can be seen in Additional file 1: Table S6 and Additional file 2: Figure S4, mRNA transcripts for some TFs are substage-specific. For example, *Pou5f1* transcript is specific to the preleptotene stage while *Zbtb33* transcript is specific to the pachytene/diplotene substages. Other TF transcripts, similar to meiosis-specific genes in general, are shared across substages but may be specific to early or late meiosis (Additional file 2: Figure S4); *Zfp143* transcript is shared across late meiosis substages while *Tpbl1* transcript is common to the preleptotene (in the “Jazf1 + 3” cluster) and late leptotene/zygotene/early pachytene (in the “Mybl* + 9” cluster) substages. We also found that some TFs, for example MYBl1, also have target genes that are negatively concordant with substages (Additional file 2: Figure S5; Additional file 1: Table S7). We used the MGI bioinformatics Batch Query tool to determine that 160 of the 181 TFs in this analysis were previously found to be highly expressed specifically and/or significantly in the testis or male germ cells and 39 are annotated to male-reproduction-related phenotypes (Additional file 1: Table S6).

To gain a deeper insight into the regulatory patterns of meiosis, we selected the highest scoring TF genes for further analysis, based on iRegulon’s NES score which corresponds to a low false discovery rate. Of these highest scoring TFs, half of them are annotated to meiosis-related functions: ETV4, E2F1, GATA2, RFX4, and ZFP143. We included four other well-known meiosis TFs: RARG, MYBL1, ETV5, and TBPL1. We compared the mRNA expression pattern of these TF genes with the expression patterns of their target genes in order to identify putative regulatory relationships, an analysis that is based on the assumption that a relevant TF protein appears more or less contemporaneously with its transcript (i.e., no translational delay). In cases where the expression pattern of the TF gene had the opposite expression pattern of its target genes, we infer that the TF acts as a repressor on these targets; but in cases where the expression patterns of the TF gene and its target genes were concordant, we postulate that the TF enhances expression of its targets. For TFs with genes that did not change over our time course, such as the known candidate NRF1, we could not infer a relationship to target genes and excluded them from further analysis.

**Fig. 4:**
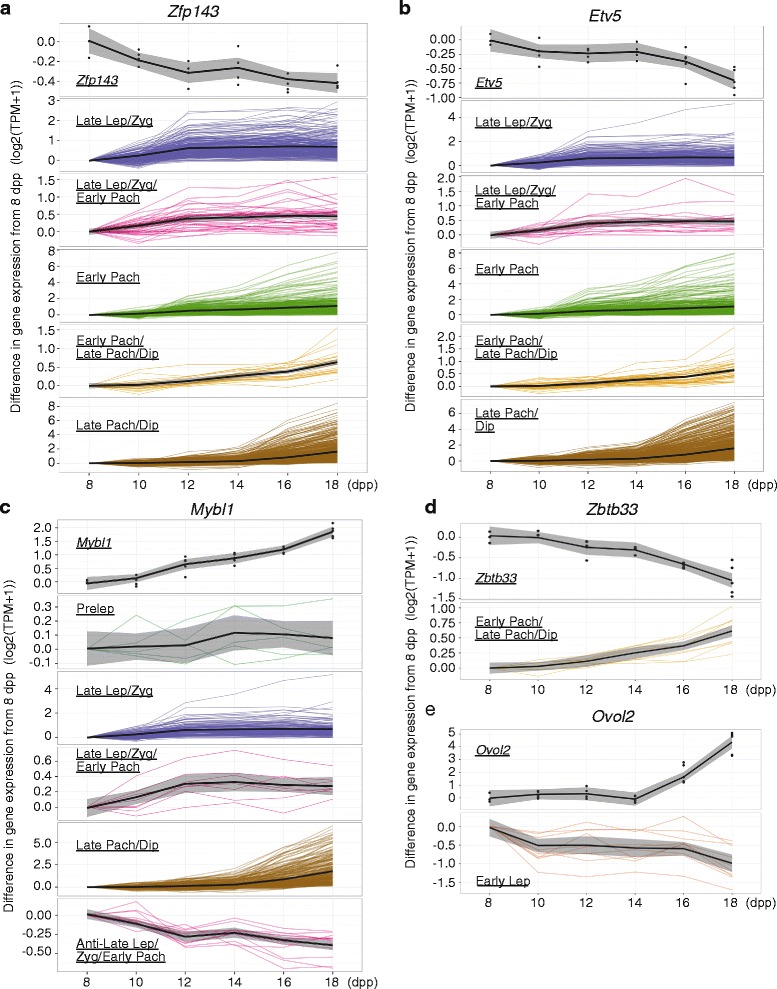
Expression of TFs and target genes for each substage. The difference from mean gene expression at 8 dpp of the TF and its target genes at each time point. To illustrate the overall pattern, a smoothed line was fit to the substage-specific gene expression. Color of lines represents the substage of target genes. Gene expression is average log2(TPM + 1), averaged over time point replicates. Expression is plotted for (**a**) *Zpf143*, (**b**) *Etv5*, (**c**) *Mybl1*, and (**d**) *Zbtb33*

**Fig. 5:**
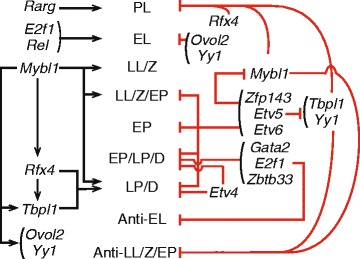
Regulation of substage-concordant gene expression during meiotic prophase. Analyses suggest that substage-concordant gene expression is regulated both positively and negatively by a network of TFs. TFs are shown by their gene names, and the meiotic substages of predicted TF target genes are shown in the center. Black arrows represent a TF acting as an enhancer on its target genes and red bars represent a TF acting as a suppressor on its target genes. (Abbreviations: PL = pre-leptonema, EL = early leptonema, LL/Z = late leptonema and zygonema, LL/Z/EP = late leptonema, zygonema, and early pachynema, EP = early pachynema, EP/LP/D = early and late pachynema and diplonema), LP/D = late pachynema and diplonema, Anti-EL = genes negatively concordant with early leptonema, Anti-LL/Z/EP = genes negatively concordant with late leptonema, zygonema, and early pachynema)

Following these assumptions, as illustrated in Figs. 4 and 5, we suggest that OVOL2 and YY1 act as repressors of target genes in early leptonema and ETV5, ETV6, and ZFP143 act as repressors on target genes after initiation of meiosis, beginning with the late leptotene and zygotene substages. Evidence suggests that ZBTB33, GATA2, and ETV4 also act as repressors on gene targets in pachynema (Fig. 4 and Additional file 2: Figure S6); while RFX4 appears to be an enhancer of target genes in late pachytene and diplotene substages (Additional file 2: Figure S6), but may repress its target genes in preleptonema, although this relationship is less clear. Interestingly, these underlying assumptions based on relative expression levels suggest that MYBL1, TBPL1, and E2F1 act as both enhancers and repressors of their target genes. MYBL1 and TBPL1 appear to repress target genes in early meiosis while enhancing target genes in late meiosis, while E2F1 has the opposite pattern and may repress target genes in late meiosis while enhancing target genes in early leptonema (Additional file 2: Figure S6). Similar regulatory switching has been shown to result from changes in protein co-factors [36] and post-translational modifications [37]. Moreover, genes for some of the TFs described above are also themselves target genes associated with specific substages. By considering these relationships, we inferred candidate regulatory interactions among TFs; for example, ZFP143 and ETV5 suppress *Mybl1* gene expression, while MYBL1 enhances expression of *Rfx4*, *Ovol2*, *Yy1*, and *Tbpl1* genes (Fig. 5).

Overall, 41 % of the genes associated with specific meiotic substages (2483 of 6052 genes) are predicted targets of one or more of these eight meiosis-related TFs. This noteworthy observation suggests that this concise regulatory network can account for a substantial portion of the meiotic program of transcription.

## Discussion

Here we identify a male meiotic germ-cell transcriptome using a novel analysis based on a dense dataset of cytological substage-specific signatures. The high concordance between RNA-seq expression data and cytological proportions of isolated germ cells across all samples we analyzed allowed us to develop a novel PMCA to identify the substage-specific transcriptomes for meiotic prophase. This computational method does not require the use of FACS or sedimentation sorting of cells and can be applied to other complex cell populations for which there are well-defined cytological criteria.

### Cytological deconvolution of RNA-seq data from the first wave of spermatogenesis

In mammalian males, spermatogenesis is continuous and asynchronous, ensuring daily capacity to deliver sperm backed up by a testis comprised of abundant numbers of germ cells at all stages of spermatogenesis. While advantageous to reproductively active males, this biological imperative has frustrated attempts to isolate stage-purified spermatogenic cells. Methods for stratification of cell samples by either sedimentation [3, 4] or flow cytometry [7, 8] enrich specific spermatogenesis cell stages, while the juvenile first wave of spermatogenesis (used in this study) provides a leading edge of differentiating cells against a background of less differentiated cells. In the laboratory mouse, meiosis is initiated at about 8 dpp in a subset of spermatogonia by retinoic acid stimulation of STRA8 [1, 20]; however, initiation of subsequent waves of meiosis make the cellular population increasingly heterogeneous (Fig. 1c), resulting in a complex histology [4]. The cell-stage frequencies presented here (Fig. 1c) are based on a powerful combinatorial application of antibodies recognizing well-characterized and highly stage-specific marker proteins. We find a continuous and relatively constant background of the earliest spermatogenic cells, namely, spermatogonia and the preleptonema and leptonema (Fig. 1c), and, not surprisingly, these “background” spermatogenic cells are the ones most refractory for computational assignment of a substage-specific transcriptome. On the other hand, pachytene spermatocytes are not present at the earliest juvenile stages, and appear in a discrete time period; consequently, it was possible to assign a robust gene list to this stage.

Gene assignments to specific substages were guided by the covariance of transcriptomic data and the cytological data. Assignment of expressed transcripts to cellular subpopulations in heterogeneous samples has long been a computational challenge [18, 38–40]. In this study, we exploited the advantage of paired RNA-seq and cytological data to develop the novel PMCA approach that isolates changes of gene expression specific to each meiotic substage. Maximum covariance analysis (MCA) was first developed in the meteorological sciences [41] and was popularized in the climatological sciences in the 1990s [42, 43]. More recently, an MCA approach has been used in a bioinformatics context to clarify relationships between gene and protein expression [44]. While MCA is often an effective tool for detecting common signals in two sets of variables, it can be limited by a tendency to fit spurious patterns when faced with increased sampling variation [45, 46]. Current methods [47] employ a parametric smoothing model using principal component regression, which requires a normality assumption or rely on GO analysis [44]. We developed a novel PMCA method that not only overcomes the spurious pattern identification liability but that does so without the need for any parametric assumptions or reliance on GO analysis. Our PMCA approach is broadly applicable to multi-dimensional data derived from a common set of samples.

### Substage specificity of meiotic gene expression and regulation

Historically, meiotic prophase substages are characterized by the morphology of chromatin, and correlated genetic mechanisms have been revealed in the past decades [48]. It is known that there is widespread transcription of protein-coding genes in the testis [19], and indeed, we found that among germ cells alone, more than 15,000 genes are transcribed, suggesting that a significant portion of testis transcriptional complexity is due to germ cells. Using PMCA, we successfully identified genes with expression patterns that matched the “cyto-patterns” of stage-specific cell frequencies determined by antibody labeling (Fig. 1c). Some genes were shared among substages; for example, late leptotene and zygotene shared 131 genes with early pachytene and early pachytene shared 106 genes with late pachytene and diplotene. Due to the strong temporal signal of contribution of the late pachytene germ cells, which do not appear until 16 dpp and greatly increase in representation by 18 dpp, we identified over 4000 late pachytene genes. However, since the proportion of preleptotene and early leptotene cells did not vary greatly over time, we were unable to deconvolve stage-specific transcriptomes for the earliest meiotic substages as confidently as for the later substages.

We compared these substage-specific gene lists to those developed in other recent analyses of the germ-cell testis transcriptome. The striking increase in number of genes unique to the pachytene stage was also observed by Soumillon, et al. [19], where, in fact, greater numbers of genes were considered expressed than in this study. However, we have used a more stringent cutoff (TPM ≥ 3) to eliminate from the data transcripts expressed at low levels. When we relaxed this requirement and compared the number of genes expressed with a cutoff of TPM > 0, we found the number of expressed transcripts to be similar to that reported by Soumillion, et al. [19]. We also compared the substage-specific gene lists to the relevant clusters identified by Margolin, et al. [18]. In their study, gene lists were temporally clustered from single samples taken at 6, 10, 12, 14, 16, 18, 20, and 38 dpp. While our study was based on multiple replicates at each time point as well as fine-grained cytological analysis, our PMCA gene lists substantially overlapped with their derived gene list clusters (Additional file 1: Table S8). In all, 4013 of the 4077 genes in clusters identified by Margolin, et al. [18] were also expressed in our dataset.

We also compared our results with those derived from flow-cytometry based methods of sorting by DNA content [5, 6]. For both of these studies, we found overall concordance in genes assigned to meiotic substages (Additional file 1: Table S9). These similarities were especially pronounced for late pachytene and diplotene substages due to the large numbers of genes in these sets. However, we also observed a broader trend of alignment between early and late substages. Furthermore, our use of multiple markers to cytologically characterize cells paired with our PMCA analysis allowed for more precise substage assignment than possible by cell sorting. For instance, our spermatogonia and pre-leptotene sets were the top two strongest overlaps with the “secondary spermatocyte” (2C) cell fraction in da Cruz, et al. [5] (hypergeometric test *p* = 4.5 x 10^-4^ and *p* = 1 x 10^-3^, respectively), but split these assignments to provide improved substage resolution. Similarly, our joint late-leptotene and zygotene genes and early pachytene genes had the two greatest overlaps with the combined “leptotene and zygotene” and “pachytene spermatocytes” (LZ + PS) genes (hypergeometric test *p* = 8.7 x 10^-7^ and *p* = 1.4 x 10^-3^, respectively), and further partition these transcripts into more precise substages. Analysis of Fallahi et al. [6] data was limited by different experimental platforms (microarrays versus RNA-seq), different assay timing (adult versus juvenile mice), and generally low numbers of uniquely assigned genes, but also revealed significant overlaps in late substages (Additional file 1: Table S9). By assessing functional concordance by similarity in GO annotations, we determined that our early leptotene and Fallahi et al. [6] pre-leptotene sets share a common enrichment in RNA transcription genes. Finally, the most prominent divergences between our data and these previous results were in the spermatogonia fraction. We note that cell sorting techniques are susceptible to inclusion of somatic-cells in this fraction, whereas our spermatogonia genes were significantly negatively-concordant with many standard meiosis genes (Additional file 1: Table S3). In sum, these comparisons suggest that our transcript sets encompass and substantially expand these previous findings.

In this study, we characterized each meiotic substage using the list of substage-specific genes from PMCA analysis. Some meiosis genes were associated to particular substages. For example, *Spata22* [49] and *Msh4/5* [50, 51] are both highly associated with late leptotene/zygotene substages, and both are required for recombination. However, many canonical meiosis genes were found throughout all meiotic prophase substages; these genes include *Rad51*, *Rec8*, and *Syce2*. This may well reflect a lack of acute transcriptional regulation for these important transcripts. Rather than transcription at the precise meiotic substage of use, quality and efficiency of meiosis may be ensured by having transcripts present and available for translation throughout meiotic prophase, with substage transitions regulated post-transcriptionally and/or post-translationally. Further, although we confirmed that spermatogonia negatively concordant genes are enriched with meiotic genes, we found several meiotic genes in the gene list concordant with spermatogonia, including *Fign*, which was reported to be required for meiotic recombination [52], suggesting that some of the meiotic program is set up prior to meiotic entry.

Not surprisingly, this developmental transcriptome analysis also revealed that many genes expressed during meiosis do not have known functions directly contributing to meiosis. These may instead be part of a parallel program of spermatogenic gene expression. For example, many of the genes expressed in early leptonema are associated with transcription or RNA metabolism, as well as with cellular processes such as cell-cell interactions, which are of considerable importance in the biology of the seminiferous tubule. PMCA also revealed that late pachytene/diplotene-expressed genes are significantly associated with GO terms associated with spermiogenic processes, correlating well with findings of other studies [5, 6]. During post-meiotic stages of nuclear condensation, transcription is globally repressed [28, 53], underlying the biological rationale for prior transcription to support the active protein synthesis during spermiogenesis. In addition, the late pachytene/diplotene-associated gene lists are significantly enriched with non-protein-coding RNAs, including lincRNAs (long intergenic noncoding RNA) and piRNA precursors.

### Meiotic regulatory networks

Analysis of predicted TF targets [35] generated a regulatory network that potentially governs the meiotic and spermatogenic programs of gene expression identified by this study (Fig. 5). The protein ZFP143 was central in our network, and is required for embryo development in zebrafish [54] and human [55, 56]. The human ortholog, ZNF143, is ubiquitously expressed [57], and binds to the promoter region of target genes where it is required for formation of chromatin loops by interacting with POLII and RAD21 [58]. Because RAD21 is differentially expressed after the pachytene stage of meiosis [59], it is possible that ZFP143 is crucial for spermatogenic processes by regulating the transcription of genes during pachytene/diplotene substage, an idea that should be tested experimentally since our analysis indicates that ZFP143 may target genes strongly associated with genes in these substages. Reinforcing the validity of our approach, our regulatory network also involved MYBL1 (also known as A-MYB). Our results support previous findings that a subset of late pachynema/diplonema-expressed genes, many involved with the spermatogenic and spermiogenic processes, are associated with MYBL1 [60, 61]. MYBL1 regulates transcription of cell cycle-related genes. Germ cells with a homozygous mutation in the *Mybl1* gene exhibit defects in meiotic chromosome synapsis [60]. Moreover, our results suggest that MYBL1 also is in a network associated with the transcription of genes encoding proteins required for piRNA biogenesis, including *Tdrd1, Tdrd6* and *Tdrd7*, consistent with previous reports [62]. Going beyond our core regulatory network, we also identified several other TFs, whose target genes include the Tdrd family and Piwi genes, potentially involved in piRNA processing: ATF3, ELF1, ELK3, ELK4, FLI1, NFYA, NFYB, NRF1, PBX3, RFX2, RFX7, SP1, YY1, ZBTB33, ZFP143 and ZFP42. This analysis not only revealed a core network for transcriptional regulation of meiotic progression (Fig. 5 and Additional file 2: Figures S4 and S5) but also suggested that a significant proportion of the genes expressed in the meiotic transcriptome may be controlled by a concise entourage of transcription factors.

## Conclusions

This study has untangled in part the complexity and parallel process of spermatogenesis and meiosis by focusing on associating gene expression with highly specific cytological signatures defining meiotic prophase substages. This unique and powerful approach to deconvolving transcriptomes of complex cell populations is applicable for discovery of transcriptional signals in other such complex cell populations or heterogeneous tissues.

## Methods

### Experimental design and mice

All C57BL/6J mice used for this study were obtained from The Jackson Laboratory (Bar Harbor, USA). All animal procedures were in accordance with the National Institute of Health guide and U.S. Department of Agriculture standards for animal care and use and were approved by the Animal Care and Use Committee at The Jackson Laboratory (Protocol #05004). Mice were euthanized at 8, 10, 12, 14, 16 and 18 days post partum (dpp) to follow the leading edge of meiotic progression during the first wave of spermatogenesis. For each time point, 5 biological replicates were sampled and germ cells were isolated from the pooled two testes of each mouse. A portion of germ cell sample was used for cytological analysis and the rest of cells were used for RNA-seq analysis. Both cytological and RNA-seq analyses were performed on all 30 samples (Fig. 1a).

### Cytological methods

#### Isolation of mixed testicular germ cells

The procedure was as previously described with some modifications [26]. Briefly, seminiferous tubules were transferred into 20 ml DMEM (Gibco, Life Technologies, Carlsbad, CA, USA) containing 0.5 mg of Liberase TL Research Grade (05401020001, Roche, Basel, Switzerland) and incubated for 20 min at 32 °C. To remove interstitial cells, tubules were washed three times with the same media. In the final wash media, the tubules were pipetted several times to form fragments, which were digested with 0.5 mg of Liberase and 10 μg of DNase in 20 ml DMEM for 13 min at 32 °C in shaking water bath. The isolated cells were further digested by pipetting for 3 min, and germ cells were isolated by filtering through Nitex mesh (53-70 μm pore size). The crude germ cells were washed three times by centrifugation for 7 min at 500 g using 10 ml of the media containing 10 μg of DNase. The cells were resuspended in 1 ml of ice-cold PBS, and cell concentration determined. 1.25 x 10^5^ cells (about 10 % of total) were used for cytological scoring, with the remainder used for RNA-seq (see below).

#### Isolation of enriched populations of adult pachytene germ cells

Each enriched population (4 biological replicates) of pachytene/diplotene spermatocytes was obtained from the testes of six 9-week-old mice by sedimentation at unit gravity [3]. Mixed germ-cell suspensions were prepared as described above, and after the three 0.5 % BSA/KRB washes, cells were separated by cellular sedimentation at unit gravity in a 2–4 % BSA gradient generated over 2.5 h in a STA-PUT apparatus (ProScience Inc., GlassShop, Toronto, ON, Canada). Following the sedimentation process, 10 ml fractions were collected and examined using light microscopy and differential interference contrast Nomarski optics. Cells were identified based on morphological criteria and size [3]. Fractions containing pachytene/diplotene spermatocytes of average purity ~90 % were pooled. For every cell separation, and for each population of cells collected, an aliquot of cells was snap-frozen for subsequent RNA extraction as described below.

#### Chromatin spread preparation

Germ-cell suspensions prepared as described above were fixed in 2 % PFA containing 0.03 % SDS and mixed with an equal volume of 0.04 % Photo-Flo (Kodak, Rochester, NY, USA). The cell suspension was applied to wells of 12-well Shandon slides, and incubated in a humid chamber for 1 h at RT. After fixation, the cells were briefly air-dried, and subjected to further fixation: 2 % PFA with SDS for 3 min and 2 % PFA without SDS for 3 min. The slides were then washed three times with 0.04 % Photo-Flo. Air-dried slides were stored at -20 °C for further use.

#### Immunostaining of spread chromatin

Spread chromatin preparations were incubated with 10 % ADB blocking solution (ADB: PBS containing 2 % BSA and 0.05 % Triton-X 100) for 10 min, the same blocking solution with SDS for 10 min, and the blocking solution without SDS. Immunolabeling was performed with rat polyclonal anti-SYCP3 (1:1000 dilution, Handel lab), mouse monoclonal anti-phosphorylated histone H2AX (1:200 dilution; 05-636, Millipore, Billerica, MA, USA), rabbit polyclonal anti-STRA8 (1:1000 dilution; ab49405, Abcam, Cambridge, England) and guinea pig polyclonal anti-H1t antibodies (1:500 dilution; Handel lab). Subsequently, secondary antibodies conjugated with Alexa 647, 594 or 488 (Molecular Probes, Invitrogen, Carlsbad, CA, USA) were used at 1:500 dilution. Nuclei were stained with DAPI (0.5 μg/mL) for 10 min, and the slides were mounted with glycerol. Images were observed using a Zeiss AxioImager.Z2 epifluorescence microscope equipped with a Zeiss AxioCam MRm CCD camera (Carl Zeiss, Jena, Germany).

### RNA methods

#### Isolation of RNA and sequencing library preparation

Isolated germ-cell samples were resuspended in QIAzol Lysis Reagent according to the manufacturer's instructions, and total RNAs were purified from homogenized cells using Qiagen RNeasy Mini Kit (74104). The quality of the isolated RNA was assessed using an Agilent 2100 Bioanalyzer instrument (Agilent Technologies, Santa Clara, CA, USA) and RNA 6000 Nano LabChip assay (5067-1511, Agilent Technologies).

The mRNA sequencing libraries were prepared using the Illumina TruSeq methodology. mRNAs were purified from total RNA using biotin tagged poly dT oligonucleotides and streptavidin coated magnetic beads. The mRNAs were then fragmented and double stranded cDNA was generated by random priming. The library was then analyzed for quality using an Agilent 2100 Bioanalyzer instrument (Agilent Technologies) and DNA 1000 LabChip assay.

#### RNA sequencing

Short 100 bp paired-end reads were generated and sequenced using an Illumina® HiSeq (Illumina, San Diego, CA, USA). Sequenced reads were filtered to keep reads for which 70 % of the base pair quality score was > 20, and the 3′ end was trimmed if the base pair quality score was < 20. Two technical replicates for each paired-end were run in different lanes and then merged.

#### RNA extraction and quantitative real-time quantitative RT-PCR

For real-time RT-PCR, total RNA was isolated from isolated germ cells or enriched germ cells (see above) using the RNeasy Mini Kit (Qiagen, Hilden, Germany), and 1 μg RNA was reverse transcribed using QuantiTect Reverse Transcription Kit (Qiagen) according to the manufacture’s instruction. The real-time RT-PCR was performed by the Applied Biosystems 7500 Real-Time PCR system (Foster City, CA. USA) using the QuntiTect SYBR Green RT-PCR kit (Qiagen). Transcript levels were normalized to the levels of *Actb* by the standard curve method [63], and are presented as the mean normalized expression in 10 μg RNA. Data are represented as mean ± estimated standard deviation. Gene-specific primers are listed in Additional file 1: Table S10.

### Computational methods

#### Alignment and expression

All RNA-seq samples were aligned with Bowtie 1.0.0 [64] and expression levels were estimated by RSEM 1.2.8 [65]. A Bowtie index was prepared for alignment to a combined (mm10) transcriptome of Ensembl Genome Reference Consortium, build 38, release 75 [66], NONCODE v4 lncRNA [25], and piRNA precursor transcripts [17]. The 214 piRNA precursors were obtained from Dr. Christian Roy. Both the NONCODE lncRNA and piRNA precursors were converted to mm10 coordinates using liftOver [67]. For this study, we used log2(TPM + 1) as the expression level, where TPM is transcripts per million, defined by RSEM [65]. A gene was deemed expressed if TPM ≥ 3 for at least one of the 28 samples.

#### Principal component analysis

To test the concordance of the expression and cytological data, independent Principal Component Analyses (PCA) were performed on each dataset using prcomp(x, scale = TRUE) in the rgl package [68] in R [69]. To produce Fig. 1d, the first and second principal components for both datasets were scaled to have the same range.

#### Permutation-based Maximum Covariance Analysis (PMCA)

We developed a novel permutation-based maximum covariance analysis (PMCA) that not only overcomes the spurious pattern identification liability of traditional maximum covariance analysis but also does not require any parametric assumptions about the data. Instead, we implemented a permutation procedure that assures the patterns are valid within a given false positive rate (FPR). To begin, let

0.1 $$ {X}_{6\times 28}\;\&\kern0.24em {Y}_{20368\times 28} $$

be the cytological and gene expression data, respectively. Then mean center each row, where $\tilde{X},\tilde{Y}$ are mean centered (Eq. 1.2.)

0.2 $$ {\tilde{x}}_{is}={x}_{is}-\frac{1}{28}{\displaystyle \sum_{s=1}^{28}{x}_{is}},\kern0.24em {\tilde{y}}_{ks}={y}_{ks}-\frac{1}{28}{\displaystyle \sum_{s=1}^{28}{y}_{ks}} $$

$$ i=1,2,\dots, 6,\kern0.24em s=1,2,\dots, 28,\kern0.24em k=1,2,\dots, 20368 $$

We compute the covariance matrix and the SVD of the covariance matrix in Eq. 0.3.

0.3 $$ {C}_{6\times 20368}=\frac{1}{28}\tilde{X}{\tilde{Y}}^T=U\varSigma {V}^T $$

Because we are interested in mapping the genes of *Y* onto substages of *X* we consider ${P}_{x\left(6\times 28\right)}={U}^T\tilde{X}$, the principal components of the covariance matrix that correspond to the substages, and calculate the homogeneous and heterogeneous regressions:

0.4 $$ {Z}_{x\left(6\times 6\right)}=X{P^T}_x,\kern0.36em {Z}_{y\left(20368\times 6\right)}=Y{P}_x^T $$

To allow for direct comparison, each row of *Z*_x_, *Z*_*y*_ is divided by its respective root mean square, Eq. 0.5.

0.5 $$ \begin{array}{l}{Z}_{x(ij)}={z}_{x(ij)}/\sqrt{{\displaystyle \sum_{j=1}^6{z}_{x(ij)}^2}/5}\\ {}{Z}_{y(kj)}={z}_{y(kj)}/\sqrt{{\displaystyle \sum_{j=1}^6{z}_{y(kj)}^2}/5}\end{array} $$

For each substage cyto-pattern (the row of *Z*_*x*_ corresponding to the substage), we find the genes that have a similar gene expression pattern across the columns of *Z*_*y*_. Through computation of the SVD, we lose a degree of freedom so the maximum number of patterns is 5; there are 6 substages. For each substage cyto-pattern, we call a gene pattern similar if it is within a certain window of the substage’s cyto-pattern.

To determine the optimal window width, we devised a permutation method that iterates through varying window widths and chooses the optimal width based on the estimated false positive rate (FPR). We stipulated that the estimated FPR be less that 0.05 by the third component (*j* = 3). Because the gene lists get finer, and more specific, as we progress through the components, the estimated FPR for cyto-patterns 4 and 5 are less that the specified 0.05. For the substage-specific gene lists described in this paper, the estimated FPR < 0.005_._ A detailed explanation of the PMCA optimal width selection is provided in Additional file 3.

### Bioinformatic analysis

#### Gene Ontology (GO) analysis

GO enrichment analysis was performed using GOrilla, Gene Ontology enRIchment anaLysis and visuaLizAtion tool [30] with ranked gene lists. Gene lists were ranked for each substage based on a score that measures how closely each gene pattern follows the substage cytopattern. GO terms was established by the GO Consortium [70] and used to group genes according to their biological or molecular functions. A total of 13,363 of the 15,025 genes expressed in our time series have at least one annotation in GO.

#### Transcription factor analyses

TFs for substage-concordant and negatively concordant genes and the TF target genes were identified using iRegulon, Version 1.3 [35] in Cytoscape, version 3.1.0 [71] with the substage-concordant and negatively concordant gene lists.

## Additional files

Additional file 1: This file contains a mini-website of Tables S1-S10. The same mini-website is available at http://carterdev.jax.org/dtx/a2/index.html. Interactive expression plots. **Table S1.** Number and proportion of isolated cells for cytological analysis and RNA-seq. **Table S2.** Gene lists and gene expression values for each of the substages, separated by worksheets. An additional worksheet provides results for X-linked genes. Abbreviations for each substage are as follows: Sp’gonia for spermatogonia, PL for preleptotene, EL for early leptotene, LL + Z for late leptotene and zygotene, LL + Z + EP for those genes found in late leptotene, zygotene, and early pachytene, EP for early pachytene, EP + LP + D for early pachytene, late pachytene, and diplotene, and LP + D for late pachytene and diplotene. Sheet names prefixed with “Anti-“correspond to genes that are negatively concordant with the substage, (e.g., Anti-PL corresponds to genes that are negatively concordant with preleptotene. Genes are ranked by how closely the expression pattern follows the substage pattern (rank = 1 is best). Gene ids and Gene names for piRNA precursors and NONCODE lncRNAs have prefix “pi-” and “NON”, respectively. Expression is given in log2(TPM + 1). **Table S3.** GO analysis for substage-negatively concordant genes. Abbreviations for each substage are as follows: Sp’gonia for spermatogonia, EL for early leptotene, LL-Z for late leptotene and zygotene, EP for early pachytene, and LP-D for late pachytene and diplotene. **Table S4.** GO analysis for substage-concordant genes. Abbreviations for each substage are as follows: Sp’gonia for spermatogonia, EL for early leptotene, LL-Z for late leptotene and zygotene, EP for early pachytene, and LP-D for late pachytene and diplotene. **Table S5.** Analysis of genes annotated to meiosis. **Table S6.** TFs for substage-concordant genes. The column names are explained as follows: “NES” is short for Normalized Enrichment Score in iRegulon, “ngenes” is the number of substage-concordant genes associated with the TF, “name” used in the arc-diagram (* indicates a family of genes, e.g., Rfx* for Rfx1, Rfx2, Rfx3, Rfx4). Bolded TFs are highly expressed in the testis and those highlighted in red are associated with male reproduction. **Table S7.** TFs for substage-negatively concordant genes. The column names are explained as follows: “NES” is short for Normalized Enrichment Score in iRegulon, “ngenes” is the number of substage negatively-concordant genes associated with the TF, “name” used in the arc-diagram (* indicates a family of genes, e.g., Rfx* for Rfx1, Rfx2, Rfx3, Rfx4). **Table S8.** Comparison of substage-concordant gene lists with derived clusters in Margolin, et al. [14]. **Table S9.** Comparison of substage-concordant gene lists with sets derived from FACS-sorted cells [5, 6]. **Table S10.** Primers for qRT-PCR. (ZIP 6513 kb) Additional file 2: This file contains a mini-website of Figures S1-S6 and a link to interactive expression plots. The same mini-website is available at http://carterdev.jax.org/dtx/a1/index.html. **Figure S1.** Expression of substage-concordant genes. (A-H) Heat maps show gene expression of substage-concordant genes at each time point. Gene expression is shown as average log2(TPM + 1) of time point replicates. **Figure S2.** Expression of substage-negatively concordant genes. (A-H) Heat maps show gene expression of substage-negatively concordant genes at each time point. Gene expression is shown as average log2(TPM + 1) of time point replicates. **Figure S3.** Validation of RNA-seq expression pattern by qRT-PCR. Box plots (top) show gene expression detected by RNA-seq at 10 dpp and 16 dpp. Bar graphs (bottom) show gene expression detected by qRT-PCR using isolated germ cells from pooled testes from 3 mice at 10 dpp and 16 dpp. Gene expression for RNA-seq is shown as log2(TPM + 1). Gene expression for qRT-PCR is shown as fold increase relative to *Actb* expression. **Figure S4.** TF arc diagram illustrating the most significant TFs across substages for the concordant gene lists. Node color represents the substage. Node size is related to significance (larger = more significant but all are highly significant with a iRegulon NES > = 4). Width of arc corresponds to how many TFs are shared between nodes (wider = more shared TFs). A node is connected to another node if a node’s TFs are a subset of the other node’s TFs. Color of the arc (degree) is related to how many connections the node has. Degree equals the number of connections. The name of each node is an abbreviation, where an asterisk indicates multiple members of the TF family are included in the node (e.g., Rfx* for Rfx1, Rfx2, Rfx3, Rfx4) and when a node name is followed by + integer, as in “Mef2* + 1”, it indicates that there are TFs in the Mef2 family plus one other TF. All TFs associated with each node are listed in Additional file 1: Table S6. The number in parenthesis next to the node name indicates the number of substage-concordant genes associated with the TF cluster. **Figure S5.** TF arc diagram illustrating the most significant TFs across substages for the negatively concordant gene lists. Node color represents the substage. Node size is related to significance (larger = more significant but all are highly significant with a iRegulon NES > = 4). Width of arc corresponds to how many TFs are shared between nodes (wider = more shared TFs). A node is connected to another node if a node’s TFs are a subset of the other node’s TFs. Color of the arc (degree) is related to how many connections the node has. Degree equals the number of connections. The name of each node is an abbreviation, where an asterisk indicates multiple members of the TF family are included in the node (e.g., Rfx* for Rfx1, Rfx2, Rfx3, Rfx4) and when a node name is followed by + integer, as in “Mef2* + 1”, it indicates that there are TFs in the Mef2 family plus one other TF. All TFs associated with each node are listed in Additional file 1: Table S7. The number in parenthesis next to the node name indicatea the number of substage-negatively-concordant genes associated with the TF cluster. **Figure S6.** Expression of TF and its target genes for each substage. The difference of mean gene expression at 8 dpp of the TF and its target genes at each time point. To illustrate the overall pattern, a smoothed line was fit to the substage-specific gene expression. Color of lines represents the substage of target genes. Gene expression is shown as average log2(TPM + 1) across time point replicates. (ZIP 13261 kb) Additional file 3: Details of permutation-based maximum covariance analysis (PMCA). (PDF 150 kb)
