# Supplementary material for: Regulatory complexity revealed by integrated cytological and RNA-seq analyses of meiotic substages in mouse spermatocytes
Source: BMC Genomics. 2016 Aug 12;17:628. doi: 10.1186/s12864-016-2865-1 (PMC4983049; doi:10.1186/s12864-016-2865-1)
Supplement: Additional file 1: — This file contains a mini-website of Tables S1-S10. The same mini-website is available at http://carterdev.jax.org/dtx/a2/index.html. Interactive expression plots. Table S1. Number and proportion of isolated cells for cytological analysis and RNA-seq. Table S2. Gene lists and gene expression values for each of the substages, separated by worksheets. An additional worksheet provides results for X-linked genes. Abbreviations for each substage are as follows: Sp’gonia for spermatogonia, PL for preleptotene, EL for early leptotene, LL + Z for late leptotene and zygotene, LL + Z + EP for those genes found in late leptotene, zygotene, and early pachytene, EP for early pachytene, EP + LP + D for early pachytene, late pachytene, and diplotene, and LP + D for late pachytene and diplotene. Sheet names prefixed with “Anti-“correspond to genes that are negatively concordant with the substage, (e.g., Anti-PL corresponds to genes that are negatively concordant with preleptotene. Genes are ranked by how closely the expression pattern follows the substage pattern (rank = 1 is best). Gene ids and Gene names for piRNA precursors and NONCODE lncRNAs have prefix “pi-” and “NON”, respectively. Expression is given in log2(TPM + 1). Table S3. GO analysis for substage-negatively concordant genes. Abbreviations for each substage are as follows: Sp’gonia for spermatogonia, EL for early leptotene, LL-Z for late leptotene and zygotene, EP for early pachytene, and LP-D for late pachytene and diplotene. Table S4. GO analysis for substage-concordant genes. Abbreviations for each substage are as follows: Sp’gonia for spermatogonia, EL for early leptotene, LL-Z for late leptotene and zygotene, EP for early pachytene, and LP-D for late pachytene and diplotene. Table S5. Analysis of genes annotated to meiosis. Table S6. TFs for substage-concordant genes. The column names are explained as follows: “NES” is short for Normalized Enrichment Score in iRegulon, “ngenes” is the number of substage-concordant [file 12864_2016_2865_MOESM1_ESM.zip › Additional file 2/TableA9.pdf]

***daCruz, et al.* [5]**	***This study***	***Best match*** ***N in common***	***p***
2C	Sp’gonia	57	0.000453
LZ	LL+Z	26	0.013907
PS	LP+D	85	6.27×10^{-18}
RS	Sp’gonia	32	0.000376
2C+LZ	EL	3	0.313099
2C+LZ+PS	EL	4	0.072829
LZ+PS	LL+Z	23	8.70×10^{-7}
LZ+PS+RS	LP+D	231	4.29×10^{-33}
PS+RS	LP+D	587	1.45×10^{-211}
2C+LZ+PS+RS	LP+D	1136	2.09×10^{-22}
LZ+RS	LL+Z	10	0.00425
2C+RS	Sp’gonia	9	0.152624
2C+PS	Sp’gonia	9	0.000204
2C+PS+RS	LP+D	65	6.20×10^{-9}
2C+LZ+RS	Sp’gonia	14	0.781228
***Fallahi, et al.* [6]**	***This study***	***N in common***	***p***
Spermatogonia	EL	1	0.096
Pre-Leptotene	Sp’gonia	2	0.45
Leptotene-Zygotene	Sp’gonia	8	0.0040
Early Pachytene	LL+Z	2	0.57
Mid Pachytene	LP+D	5	0.0074
Late Pachytene	LP+D	16	3.6×10^{-9}
Diplotene	LP+D	9	1.7×10^{-5}

Table A9. Correspondence between PMCA-derived concordant gene sets and gene sets in da Cruz, *et al*. [5] and Fallahi, *et al.* [6]. Best match determined by greatest enrichment of overlapping transcripts (lowest *p*-values, hypergeometric tests). Common transcripts from Fallahi, *et al.* were assessed by assigning the top 50 microarray probes unique to each substage to transcripts that also appeared in our RNA-seq data, which severely limited concordance analysis to only tens of genes.
