# Supplementary material for: Regulatory complexity revealed by integrated cytological and RNA-seq analyses of meiotic substages in mouse spermatocytes
Source: BMC Genomics. 2016 Aug 12;17:628. doi: 10.1186/s12864-016-2865-1 (PMC4983049; doi:10.1186/s12864-016-2865-1)
Supplement: Additional file 2: — This file contains a mini-website of Figures S1-S6 and a link to interactive expression plots. The same mini-website is available at http://carterdev.jax.org/dtx/a1/index.html. Figure S1. Expression of substage-concordant genes. (A-H) Heat maps show gene expression of substage-concordant genes at each time point. Gene expression is shown as average log2(TPM + 1) of time point replicates. Figure S2. Expression of substage-negatively concordant genes. (A-H) Heat maps show gene expression of substage-negatively concordant genes at each time point. Gene expression is shown as average log2(TPM + 1) of time point replicates. Figure S3. Validation of RNA-seq expression pattern by qRT-PCR. Box plots (top) show gene expression detected by RNA-seq at 10 dpp and 16 dpp. Bar graphs (bottom) show gene expression detected by qRT-PCR using isolated germ cells from pooled testes from 3 mice at 10 dpp and 16 dpp. Gene expression for RNA-seq is shown as log2(TPM + 1). Gene expression for qRT-PCR is shown as fold increase relative to Actb expression. Figure S4. TF arc diagram illustrating the most significant TFs across substages for the concordant gene lists. Node color represents the substage. Node size is related to significance (larger = more significant but all are highly significant with a iRegulon NES > = 4). Width of arc corresponds to how many TFs are shared between nodes (wider = more shared TFs). A node is connected to another node if a node’s TFs are a subset of the other node’s TFs. Color of the arc (degree) is related to how many connections the node has. Degree equals the number of connections. The name of each node is an abbreviation, where an asterisk indicates multiple members of the TF family are included in the node (e.g., Rfx* for Rfx1, Rfx2, Rfx3, Rfx4) and when a node name is followed by + integer, as in “Mef2* + 1”, it indicates that there are TFs in the Mef2 family plus one other TF. All TFs associated with each node are listed in Additional file 1: Table S6. [file 12864_2016_2865_MOESM2_ESM.zip › Additional file 1/FigureA1.pdf]

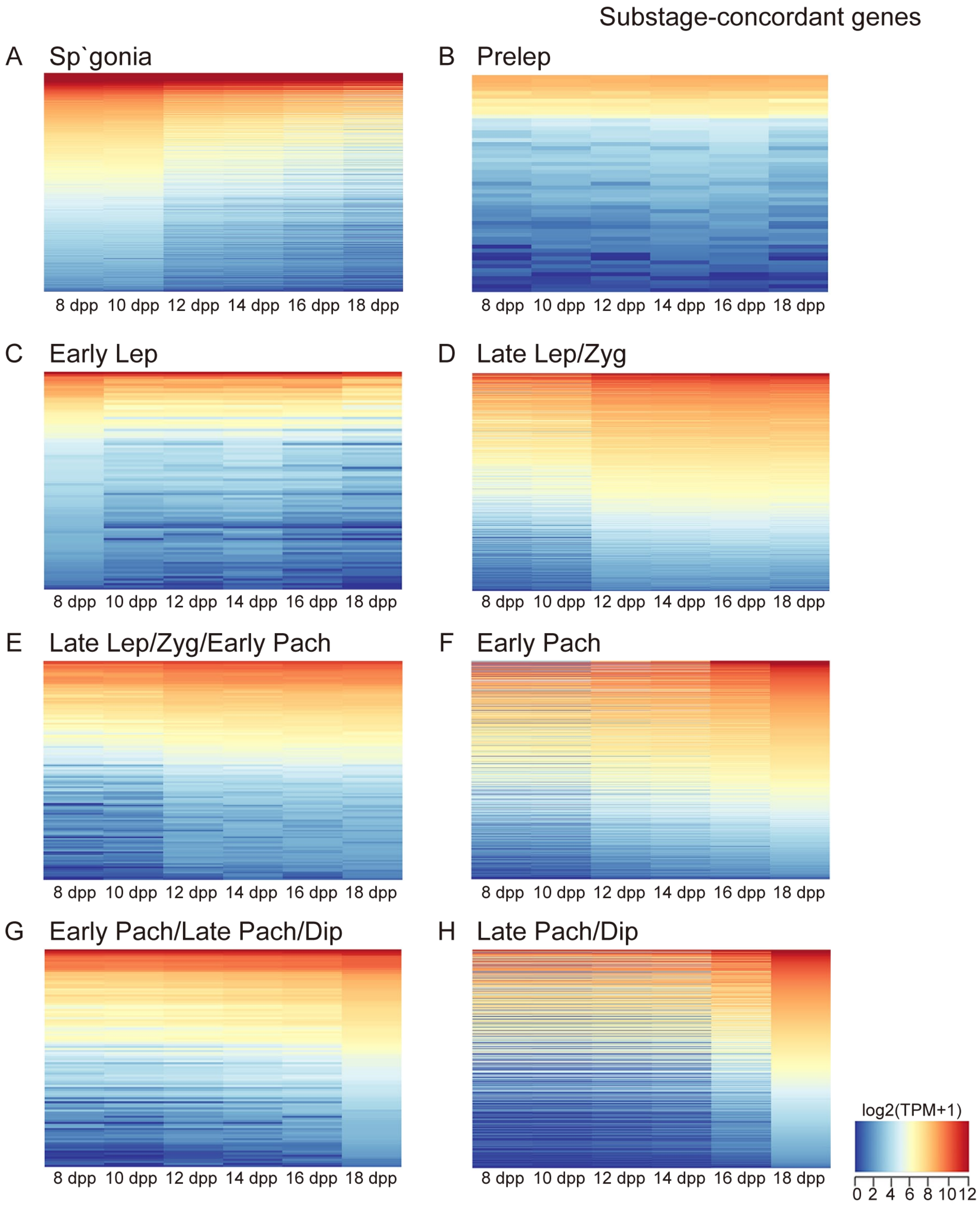
Substage-concordant genes
A Sp`gonia
B Prelep
C Early Lep
D Late Lep/Zyg
E Late Lep/Zyg/Early Pach
F Early Pach
G Early Pach/Late Pach/Dip
H Late Pach/Dip
8 dpp 10 dpp 12 dpp 14 dpp 16 dpp 18 dpp
log2(TPM+1)
0 2 4 6 8 10 12
