# Supplementary material for: Regulatory complexity revealed by integrated cytological and RNA-seq analyses of meiotic substages in mouse spermatocytes
Source: BMC Genomics. 2016 Aug 12;17:628. doi: 10.1186/s12864-016-2865-1 (PMC4983049; doi:10.1186/s12864-016-2865-1)
Supplement: Additional file 3: — Details of permutation-based maximum covariance analysis (PMCA). (PDF 150 kb) [file 12864_2016_2865_MOESM3_ESM.pdf]

Additional file 3 for
"Regulatory complexity during meiotic substages revealed by coupled cytological and RNA-seq analyses of mouse spermatocytes"
R.L. Ball, Y. Fujiwara, F. Sun, J. Hu, M. Hibbs, M.A. Handel, and G.W. Carter
***BMC Genomics*, 2016**

1 Details of permutation-based maximum covariance analysis (PMCA)

Recall that there are 28 biological samples, 6 substages, and 20368 expressed transcripts. Let $\boldsymbol{X}_{(6\times 28)}$ represent the substage cytological proportions of each sample and $\boldsymbol{Y}_{(20368\times 28)}$ represent the gene expression of each sample. We wanted to know what genes of $\boldsymbol{Y}$ have the same expression pattern as the meiotic substages of $\boldsymbol{X}$; that is, we map genes onto substages.

$$\boldsymbol{X} = \begin{bmatrix} x_{11} & x_{12} & \cdots & x_{1,28} \\ x_{21} & x_{22} & \cdots & x_{2,28} \\ \vdots & \vdots & \vdots & \vdots \\ x_{61} & x_{62} & \cdots & x_{6,28} \end{bmatrix}, \boldsymbol{Y} = \begin{bmatrix} y_{11} & y_{12} & \cdots & y_{1,28} \\ y_{21} & y_{22} & \cdots & y_{2,28} \\ \vdots & \vdots & \vdots & \vdots \\ y_{20368,1} & y_{20368,2} & \cdots & y_{20368,28} \end{bmatrix}$$

First, center the matrices by substracting the row averages. For $i = 1, 2, \ldots 6, k = 1, 2, \ldots 20, 368, s = 1, 2, \ldots 28$.

$$\begin{aligned} \tilde{x}_{is} &= x_{is} - \frac{1}{28}\sum_{s=1}^{28} x_{is} \\ \tilde{y}_{ks} &= y_{ks} - \frac{1}{28}\sum_{s=1}^{28} y_{ks} \end{aligned} \tag{1}$$

so that

$$\tilde{\boldsymbol{X}} = \begin{bmatrix} \tilde{x}_{11} & \tilde{x}_{12} & \cdots & \tilde{x}_{1,28} \\ \tilde{x}_{21} & \tilde{x}_{22} & \cdots & \tilde{x}_{2,28} \\ \vdots & \vdots & \vdots & \vdots \\ \tilde{x}_{61} & \tilde{x}_{62} & \cdots & \tilde{x}_{6,28} \end{bmatrix}, \tilde{\boldsymbol{Y}} = \begin{bmatrix} \tilde{y}_{11} & \tilde{y}_{12} & \cdots & \tilde{y}_{1,28} \\ \tilde{y}_{21} & \tilde{y}_{22} & \cdots & \tilde{y}_{2,28} \\ \vdots & \vdots & \vdots & \vdots \\ \tilde{y}_{20368,1} & \tilde{y}_{20368,2} & \cdots & \tilde{y}_{20368,28} \end{bmatrix}$$

Let $\boldsymbol{C}_{(6\times 20368)} = \tilde{\boldsymbol{X}}\tilde{\boldsymbol{Y}}^T/28$ be the covariance matrix of $\tilde{\boldsymbol{X}}$ and $\tilde{\boldsymbol{Y}}$ and use singular value decomposition (SVD) to decompose $\boldsymbol{C} = \boldsymbol{U}\boldsymbol{\Sigma}\boldsymbol{V}^T$.

Then, the column vectors of $\boldsymbol{U}$ correspond to the structures in the cytological data, $\boldsymbol{X}$, that explain the covariance and the column vectors of $\boldsymbol{V}$ correspond to the structures in the gene expression data, $\boldsymbol{Y}$, that explain the covariance.

Up to this point, PMCA is consistent with traditional MCA but the following modifications are novel.

Because we are primarily interested in mapping the genes of $\boldsymbol{Y}$ onto the substages of $\boldsymbol{X}$, we consider $\boldsymbol{P}_{x(6\times 28)} = \boldsymbol{U}^T\tilde{\boldsymbol{X}}$, the principal components of the covariance matrix that correspond to the elements of $\boldsymbol{X}$, and calculate the homogeneous and heterogeneous regressions using $\boldsymbol{P}_x$.

Notice that the last column of $\boldsymbol{P}_x = \boldsymbol{0}$.

The cytological amplitudes, or "cytopatterns", of $\boldsymbol{X}$ are

$$\begin{aligned}\boldsymbol{Z}_{x(6\times 6)} &= \boldsymbol{X}\boldsymbol{P}_x^T \\ &= \begin{bmatrix} z_{x11} & z_{x12} & \cdots & z_{x16} \\ z_{x21} & z_{x22} & \cdots & z_{x26} \\ \vdots & \vdots & \vdots & \vdots \\ z_{x61} & z_{x62} & \cdots & z_{x66} \end{bmatrix}\end{aligned}$$

and the gene amplitudes, or "gene patterns", of $\boldsymbol{Y}$ are

$$\begin{aligned}\boldsymbol{Z}_{y(20368\times 6)} &= \boldsymbol{Y}\boldsymbol{P}_x^T \\ &= \begin{bmatrix} z_{y11} & z_{y12} & \cdots & z_{y16} \\ z_{y21} & z_{y22} & \cdots & z_{y26} \\ \vdots & \vdots & \vdots & \vdots \\ z_{y20368,1} & z_{y20368,2} & \cdots & z_{y20368,6} \end{bmatrix}\end{aligned}$$

To put $\boldsymbol{Z}_x$ and $\boldsymbol{Z}_y$ on the same scale, divide each row by its respective root mean square.

For $i = 1, 2, \ldots, 6, j = 1, 2, \ldots, 6$, and $k = 1, 2, \ldots 20368$

$$z_{xij} = z_{xij} \Big/ \sqrt{\sum_{j=1}^{6} z_{xij}^2/(6-1)} \qquad (2)$$

$$z_{ykj} = z_{ykj} \Big/ \sqrt{\sum_{j=1}^{6} z_{ykj}^2/(6-1)} \qquad (3)$$

A measure of similarity between the amplitudes in $\boldsymbol{Z}_x$ and the amplitudes in $\boldsymbol{Z}_y$ is the absolute difference, or score:

$$\text{score}_{ikj} = |z_{xij} - z_{ykj}| \qquad (4)$$

Now, we can directly compare $\boldsymbol{Z}_x$ and $\boldsymbol{Z}_y$ using the scores: we say the cytopattern of ith substage of $\boldsymbol{Z}_x$ is *similar* to the gene pattern in kth gene of $\boldsymbol{Z}_y$ if the score$_{ikj}$ is small across all $p-1$ components. It is not enough to calculate the total score because we need the pattern to match across all components.

We must specify how similar is similar across the $p-1$ components while controlling for false positives. To choose the optimal window widths for the $p-1$ components, we use an iterative permutation procedure.

1.1 Permutation procedure

To avoid the traditional liability of identifying spurious patterns from the data, we estimate the false positive rate (FPR) by breaking the relationship between $\boldsymbol{X}$ and $\boldsymbol{Y}$. (Note: we chose $B = 1000$.)

for b in 1 to B (B large) {

independently shuffle the columns of $\boldsymbol{X} \rightarrow \boldsymbol{X}_b^*$

calculate $\boldsymbol{Z}_x^*, \boldsymbol{Z}_y^*$ and score$^*{}_{(ikj)b}$ using $\boldsymbol{X}_b^*, \boldsymbol{Y}$

}

Now that we have a set of scores indicative of no relationship between $\boldsymbol{X}$ and $\boldsymbol{Y}$, we use an iterative procedure to determine optimal window widths for the $j = 1, 2, \ldots, 5$ components (columns of $\boldsymbol{Z}_x, \boldsymbol{Z}_y$).

1.2 Iterative procedure

To compare $\boldsymbol{Z}_x, \boldsymbol{Z}_y$, we have to determine the optimal window widths for the 5 components while controlling the FPR; we use the scores* calculated in the permutation procedure.

Set $\tau = 1$ and let $\hat{\sigma}_j$ be the estimated standard deviation of column j of $\boldsymbol{Z}_x$. We optimize the window width by iteratively increasing τ (narrowing the width of the window).

Since $\boldsymbol{X}$ is permuted, choose $i = 1$ (i doesn't matter).
Specify α and $J \in 1, 2, \ldots, 5$ such that the estimated FPR $\leq \alpha$ by component J. We chose $\alpha = 0.05$ and $J = 3$.

Step 1:
$\boldsymbol{w} = [\hat{\sigma}_1, \hat{\sigma}_2, \ldots, \hat{\sigma}_5]/\tau$

for b in 1 to B {

$g_1 = \{$all k rows of $\boldsymbol{Z}^*_{yk1}$ for which score$^*{}_{(1k1b)} \leq w_1\}$

calculate $\hat{\text{FPR}}_{1b} = |g_1|/20368$

for j in 2 to 5 {
$g_j = g_{j-1} \cap \{$all k rows of $\boldsymbol{Z}^*_{ykj}$ for which score$^*{}_{(1kj)b} \leq w_j\}$
calculate $\hat{\text{FPR}}_{jb} = |g_j|/20368$ }

}

Step 2:
for j in 1 to 5 {

calculate the estimated FPR for component j : $\hat{\text{FPR}}_j = \sum_{b=1}^{B} \hat{\text{FPR}}_{jb}/B$

}
if $\hat{\text{FPR}}_J \leq \alpha$, STOP
else, $\tau = \tau + .1$. Go to Step 1.

The optimal window width is the final $\boldsymbol{w}_{\text{opt}}$. Use the values of $\boldsymbol{w}_{\text{opt}}$ to do the same procedure for the real data, $\boldsymbol{Z}_x, \boldsymbol{Z}_y$, and map genes of $\boldsymbol{Y}$ onto the substages of $\boldsymbol{X}$ (see subsection 1.3). Note that by taking the mean, the estimated FPR is a conservative estimate, as seen in Fig. 1.

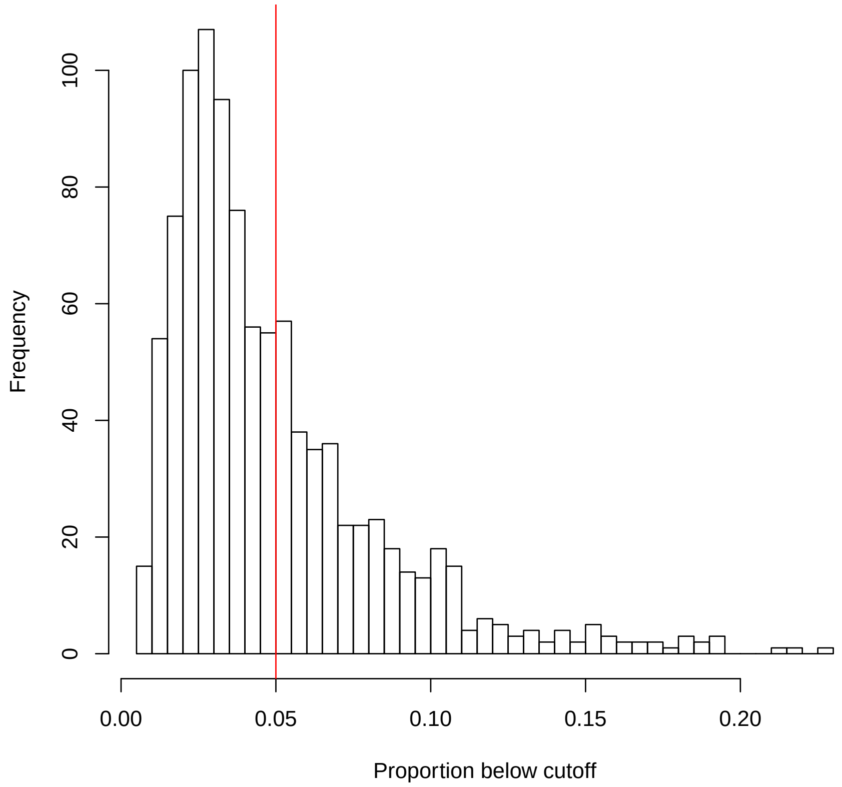

Figure 1: Histogram of the estimated FPR for the late pachytene and diplotene substage at $j = 3$. The red line is $\alpha = .05$.

The resulting estimated FPRs for all 5 components are given in Table 1.

component (j)	1	2	3	4	5
$\hat{\text{FPR}}$	0.482	0.164	0.049	0.005	<0.001
$\boldsymbol{w}_{\text{opt}}$	1.435	0.694	0.544	0.168	0.063

Table 1: Estimated FPRs and optimal window widths.

1.3 Map genes of $\boldsymbol{Y}$ onto substages of $\boldsymbol{X}$

Using the optimal window width, $\boldsymbol{w}_{\text{opt}}$, calculated above, map genes of $\boldsymbol{Y}$ onto substages of $\boldsymbol{X}$.

for i in 1 to 6 {

$g_{i1} = \{$all k rows of $\boldsymbol{Z}_{yk1}$ for which $\text{score}_{ik1} \leq w_{\text{opt}1}\}$

for j in 2 to 5 {

$g_{ij} = g_{i,j-1} \cap \{$all k rows of $\boldsymbol{Z}_{ykj}$ for which $\text{score}_{ikj} \leq w_{\text{opt}j}\}$

}

```
}
```

It is straightforward to also determine those genes of $\boldsymbol{Y}$ that are negatively concordant with substages of $\boldsymbol{X}$. Just use $-\boldsymbol{Z}_x, \boldsymbol{Z}_y$ in the above procedure. Negatively concordant elements will have the exact opposite pattern; when the cytological proportion of the substage is high, the gene expression is low, and vice versa. The resulting concordant and negatively concordant substage-specific gene lists are based on the results of $j = 4, 5$ with $\hat{\text{FPR}} \leq 0.005$.
